# A Genome-Wide Association Study of Neuroticism in a Population-Based Sample

**DOI:** 10.1371/journal.pone.0011504

**Published:** 2010-07-09

**Authors:** Federico C. F. Calboli, Federica Tozzi, Nicholas W. Galwey, Athos Antoniades, Vincent Mooser, Martin Preisig, Peter Vollenweider, Dawn Waterworth, Gerard Waeber, Michael R. Johnson, Pierandrea Muglia, David J. Balding

**Affiliations:** 1 Department of Epidemiology and Biostatistics, Imperial College London, London, United Kingdom; 2 Genetics Division, Drug Discovery, GlaxoSmithKline R&D, Verona, Italy; 3 Discovery Biometrics Respiratory, GlaxoSmithKline, Greenford, United Kingdom; 4 Department of Computer Science, University of Cyprus, Nicosia, Cyprus; 5 Genetics Division, Drug Discovery, GlaxoSmithKline R&D, Upper Merion, Pennsylvania, United States of America; 6 Department of Psychiatry, University Hospital Center and University of Lausanne, Lausanne, Switzerland; 7 Department of Internal Medicine, University Hospital Center and University of Lausanne, Lausanne, Switzerland; 8 Department of Clinical Neurosciences, Imperial College London, London, United Kingdom; 9 Discovery Medicine, Neurosciences CEDD, GlaxoSmithKline R&D, Verona, Italy; 10 Genetics Institute, University College London, London, United Kingdom; University of Muenster, Germany

## Abstract

Neuroticism is a moderately heritable personality trait considered to be a risk factor for developing major depression, anxiety disorders and dementia. We performed a genome-wide association study in 2,235 participants drawn from a population-based study of neuroticism, making this the largest association study for neuroticism to date. Neuroticism was measured by the Eysenck Personality Questionnaire. After Quality Control, we analysed 430,000 autosomal SNPs together with an additional 1.2 million SNPs imputed with high quality from the Hap Map CEU samples. We found a very small effect of population stratification, corrected using one principal component, and some cryptic kinship that required no correction. *NKAIN2* showed suggestive evidence of association with neuroticism as a main effect (*p*<10^−6^) and *GPC6* showed suggestive evidence for interaction with age (*p*≈10^−7^). We found support for one previously-reported association (*PDE4D*), but failed to replicate other recent reports. These results suggest common SNP variation does not strongly influence neuroticism. Our study was powered to detect almost all SNPs explaining at least 2% of heritability, and so our results effectively exclude the existence of loci having a major effect on neuroticism.

## Introduction

Neuroticism is a personality trait that has been associated with several psychiatric disorders, and is considered one of the risk factors for the development of depression, anxiety disorders [1], [2], [3], [4] and dementia [5]. When measured by the Eysenk Personality Questionnaire (EPQ) [6], neuroticism has been shown to be relatively stable, with a stability of 0.62 over 20 years [7], although there is some evidence of a moderate decline in older ages [8], [9]. Neuroticism is consistently higher in females than males [10], [11], but it is unclear whether this is a direct effect of gender or a consequence of different environments experienced by males and females [8], [12]–[14]. The heritability of neuroticism has been estimated in the range 0.30 to 0.50, based on twin studies [7], [15]–[17] that also show a genetic covariance of neuroticism with anxiety and depression [10], [13], [18]–[22].

Significant or suggestive linkage has been reported within regions on chromosomes 1p, 1q, 4q, 6p, 7p, 11p, 11q, 12q, 13q and 15q [23]–[26]. Shifman [27] reported a genome-wide association study for EPQ-N in 2,054 extreme-scoring individuals from a large cohort (patient registers of UK general practitioners) using eight DNA pools. No significant SNPs were identified under linkage peaks derived from sib pairs in the same cohort [14], [23]. They reported an association at SNP rs702543, in the phosphodiesterase 4D gene (*PDE4D*) on chromosome 5q, but even after individual genotyping of a further 1534 individuals from the same cohort, the combined p-value was not genome-wide significant (*p* = 2×10^−6^). Further analysis of rs702543 in a combined sample from three populations (Australian twins, Virginia Adult Twin registry, Netherlands twin families), using a measure of neuroticism that is similar to EPQ-N, showed a non-significant trend for an excess of the risk allele (A) in high neuroticism-score individuals. A family-based analysis revealed overtransmission of the A allele in high-score individuals from the Australian sample (*p* = 0.04), but not in the Netherlands sample. Thus, the association of rs702543 with neuroticism is notable but requires confirmation. In a genome-wide study of US-based and German samples, van den Oord [28] found association between neuroticism and four SNPs in the chr 14 gene *MAMDC1* (combined *p*<10^−6^). To date, the most recent association study [29] failed to replicate an association between the candidate gene *NPY* and stress response and emotion (reported by Zhou [30], using instruments other than the EPQ).

We conducted the largest genome-wide association study to date, with 2,235 individuals from a population-based collection, for whom EPQ-N score and 430K SNP genotypes were available. We also analysed an additional 1.2 M well-imputed SNPs.

## Results

The sample consists of individuals who previously participated in the ‘CoLaus’ study [31] a community survey of 6 738 randomly-selected residents of the city of Lausanne (Switzerland). Of the 3 400 Caucasians who accepted the psychiatric evaluation (55% participation rate), 2 239 individuals also completed and returned the EPQ questionnaire (66% return rate), and had genotype data available.

The mean age of the study individuals was 51.9 (SD 8.85; range 36–70), and 55.4% were female. These compare with mean age 51.2 (SD 8.82; range 36–70) and 53.0% female among all 3,307 PsyCoLaus individuals, indicating that responders to the questionnaire are slightly older and more likely to be female than non-responders.

**Figure 1:**
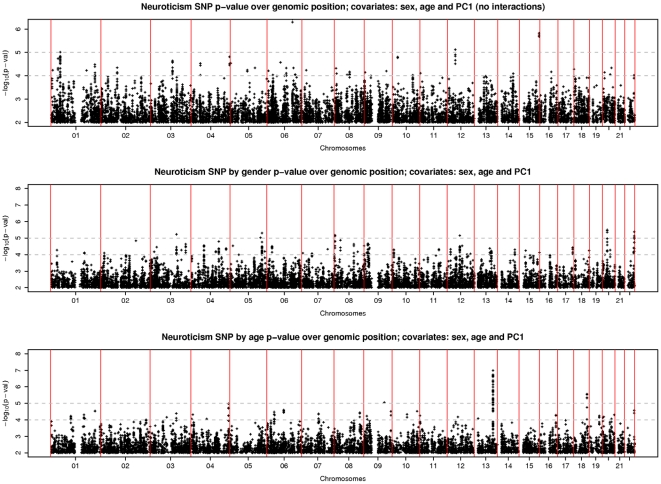
P-values over genomic location. -log_10_(p-value) for association with EPQ-N, against genomic location for 1.7 M genotyped and well-imputed SNPs. Top: main SNP effect; middle: sex by SNP interaction; bottom: age by SNP interaction.

**Figure 2:**
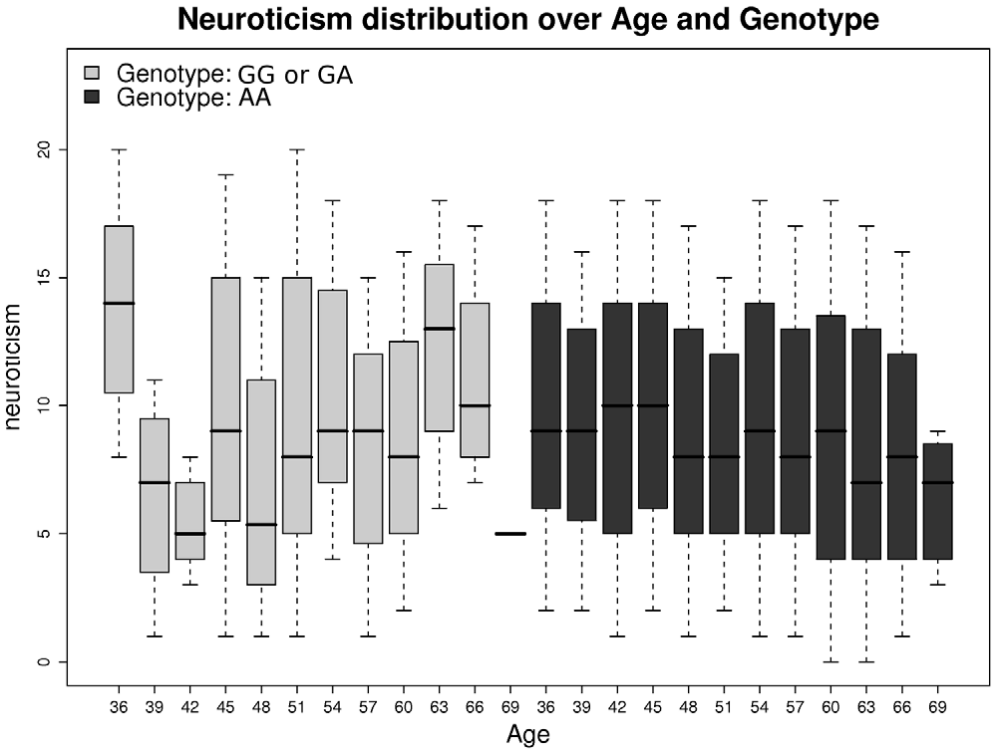
EPQ-N distribution by 3-year age groups according to minor allele carrier status at rs9561329.

A QQ plot of the *p*-values for the genotyped SNPs (supplementary material File S1) suggested negligible inflation due to population structure or cryptic relatedness (genomic control λ = 1.014), but also little evidence of *p*-values below those expected under the null hypothesis. Results from the main-effects analyses are shown in Figure 1a, and Table 1. Both genotyped and imputed SNPs within the gene *ARRDC4*, at the distal end of 15q, almost reached *p* = 10^−6^. We identified several imputed SNPs with *p*<10^−6^ on 6q, within the gene *NKAIN2*, which has not previously been reported in linkage or association studies, although the strongest association at a genotyped SNP (rs11154221) in this gene has *p*≈10^−4^. No SNP showed a notable interaction with sex (Figure 1b). SNP by age interaction analyses revealed multiple associations at the distal end of 13q within gene *GPC6* (Figure 1c, Table 1). The strongest signal (p≈10^−7^) was at a genotyped SNP, rs9561329; EPQ score tends to increase with age for carriers of the minor allele, and decreases for non-carriers (Figure 2). Conditional regression analyses revealed that the signal at each of the SNPs reported in Table 1 effectively disappeared when the most strongly-associated SNP at that locus was included as a predictor in the regression, suggesting at most one causal variant underlying each signal.

**Table 1:** Most significant main effect and interaction results for association with neuroticism (EPQ-N).

SNP main effect, *p*<2.5×10^−6^							
chr	position	snp	-log_10_(*p-val*)	Beta (SE)	gene	Alleles (MAF)	genotyped?
6	q21	rs12204812	6.43	−0.91 (0.18)	NKAIN2	A/C (0.33)	
6	q21	rs9491140	6.47	−0.91 (0.18)	NKAIN2	C/T (0.33)	
6	q21	rs9491142	6.45	−0.91 (0.18)	NKAIN2	A/G (0.33)	
6	q21	rs12192208	6.29	−0.90 (0.18)	NKAIN2	A/C (0.33)	
15	q26.3	rs1043372	5.68	−0.82 (0.17)	ARRDC4	C/T (0.38)	Y
15	q26.3	rs1043374	5.75	−0.83 (0.17)	ARRDC4	C/A (0.38)	Y
15	q26.3	rs4965121	5.83	−0.84 (0.17)	ARRDC4	C/G (0.38)	

SNP by Age, *p*<2.5×10^−7^							
chr	position	snp	-log_10_(*p-val*)	Beta (SE)	gene	Alleles (MAF)	genotyped?
13	q32	rs2025947	6.74	−0.18 (0.03)	GPC6	C/T (0.9)	
13	q32	rs3814277	6.60	−0.17 (0.03)	GPC6	C/G (0.1)	
13	q32	rs8002366	6.68	−0.17 (0.03)	GPC6	C/T (0.09)	
13	q32	rs9561326	6.64	−0.17 (0.03)	GPC6	C/T (0.09)	
13	q32	rs9561329	6.98	−0.17 (0.03)	GPC6	A/G (0.09)	Y
13	q32	rs9301877	6.72	−0.18 (0.03)	GPC6	A/G (0.09)	

SNP rs702543 in gene *PDE4D*, reported by Shifman [27] to be associated with neuroticism, was not present among our 1.7 M genotyped or imputed SNPs. However, we obtained *p* = 0.006 for association at the imputed SNP rs296410 that showed the highest R^2^ with rs702543 in the Hap Map CEU samples (R^2^ = 0.51); in our study, the major allele (T, frequency 0.52) at rs296410 shows a positive linear association with EPQ-N score. We could not replicate the association found by van den Oord [28] for the gene *MAMDC1* on 14q. Three of the four SNPs they reported had a perfect proxy in our SNP set, but our lowest *p*-value in this gene was *p* = 0.43. We tested 3 genotyped SNPs in the gene *NPY* studied by Zhou^30^, including a SNP, rs16126, in high LD (R^2^≈0.9) with two of their reported SNPs, but we found *p*>0.4 at all three SNPs. Finally, we found no significant enrichment of a GO functional category among SNPs with *p*<10^−4^.

A separate analysis was carried out on the X chromosome, including the pseudo-autosomal region. Genotypes were coded as 0, 1, and 2 for females, and 0 and 2 for males, and sex was included as a covariate in the linear regression model. No significant associations were found.

## Discussion

We present a comprehensive analysis of a population-based sample for genome-wide association with neuroticism. The evidence from 19 cryptic first-degree relative pairs in our study suggests a heritability for EPQ-N of at most 0.22, lower than the previously-reported range of 0.3 to 0.5. We identified SNPs in *NKAIN2* showing evidence for an additive main effect, but short of genome-wide significance. This gene has been linked with neurological phenotypes [41], though little functional or molecular information is available. Several SNPs in gene *GPC6* on 13q gave a signal for an interaction with age. *GPC6* is known to be involved in the regulation of the *Wnt* pathway [42] that plays a role in intracellular signalling both during neural development [43] and also in the mature nervous system with possible implication in synaptic modulation and plasticity. There is some evidence that abnormalities of signal transduction may be implicated in the pathophysiology of psychiatric disorders, including mood disorders and schizophrenia [44], [45]. Several studies also suggest that Wnt-pathway abnormalities may play a role in neurodegenerative process and Alzheimer disease [46], [47]. Neuroticism, alone or in combination with other personality traits or lifestyle, has been shown to be predictive for Alzheimer [5], [48].

It is difficult to speculate why our novel, population-based heritability estimate is so much lower than those from previous family- and studies. Common environment may partly explain a familial effect that may have been misattributed to genetics. Like all studies, ours has a number of limitations. The EPQ-N was assessed from self-administered questionnaires posted back to the researchers. Only 66% of the eligible subjects sent back the questionnaire, which may have generated a response bias, although we have shown that there are only minor differences in sex and age between responders and non-responders. Our study has a cross-sectional design, and individuals suffering a neurological condition such as dementia may be less likely to complete and return the EPQ. However, all participants in the PsyCoLaus had a psychiatric assessment and completed a Mini Mental Stage Examination [49]. If a locus has pleiotropic effects, affecting both neuroticism and dementia in old age, this could generate an age-dependent response-bias towards protective alleles, leading to a genotype by age interaction such as we observed at GPC6.

Our study was powered to detect almost all **S**NPs explaining at least 2% of phenotypic variation, and so our results effectively exclude the hypothesis that there are major loci affecting neuroticism in the Lausanne population. The lack of strong genetic association with EPQ-N score might imply that neuroticism is controlled by many loci each of relatively small effect, as is the case for many other complex phenotypes. There may also be important environmental interactions with risk factors not available in our study. If confirmed by additional studies, the suggestive age by genotype interaction that we report raises interesting methodological and biological questions.

## Materials and Methods

### Sample and assessment

The CoLaus was designed to assess cardiovascular disease risk factors and to collect plasma samples for the study of genetic variants and biomarkers. The random sampling procedure was based on a list of Lausanne inhabitants aged 35–75 years (n = 56 694 in 2003), obtained from the population register of the city. From the original study, all 35 to 66 year-old participants were invited to participate in the psychiatric arm of the study; for more details on the rationale, design and methods of the study see Preisig [32]. All subjects who were sufficiently fluent in French or English and agreed to participate were included into the PsyCoLaus sub-study and underwent the psychiatric assessment between 2004 and 2008. The present analysis was restricted to Caucasians (92% of the sample). The study was approved by the Local Ethics Committee and was supported in part by GlaxoSmithKline. All participants were duly informed about this sponsorship, and consented for the use of biological samples and data by GlaxoSmithKline and its subsidiaries.

All the participants were interviewed using the Diagnostic Interview for Genetic Studies [33] (DIGS), in order to formulate DSM-IV and subthreshold diagnoses. Additional assessment included: medical records, family history [Family History-Research Diagnostic Criteria (FH-RDC) developed by Andreasen [34]], several self-report questionnaires including EPQ and STAI, and life events interview.

The EPQ is a 90-item self-report personality inventory [6] that has been widely used for personality assessment in psychiatry and other medical fields. Factor analysis of both English and French versions revealed four dimensions that have been labelled Extraversion, Neuroticism, Psychoticism, and Social desirability (Lie scale). Reliabilities of the resulting scales, and correlations between factors, were found to be similar between English and French versions. In particular, using three different French samples, Eysenck [6] reported Cronbach alpha coefficients of 0.78 to 0.87 for neuroticism.

**Figure 3:**
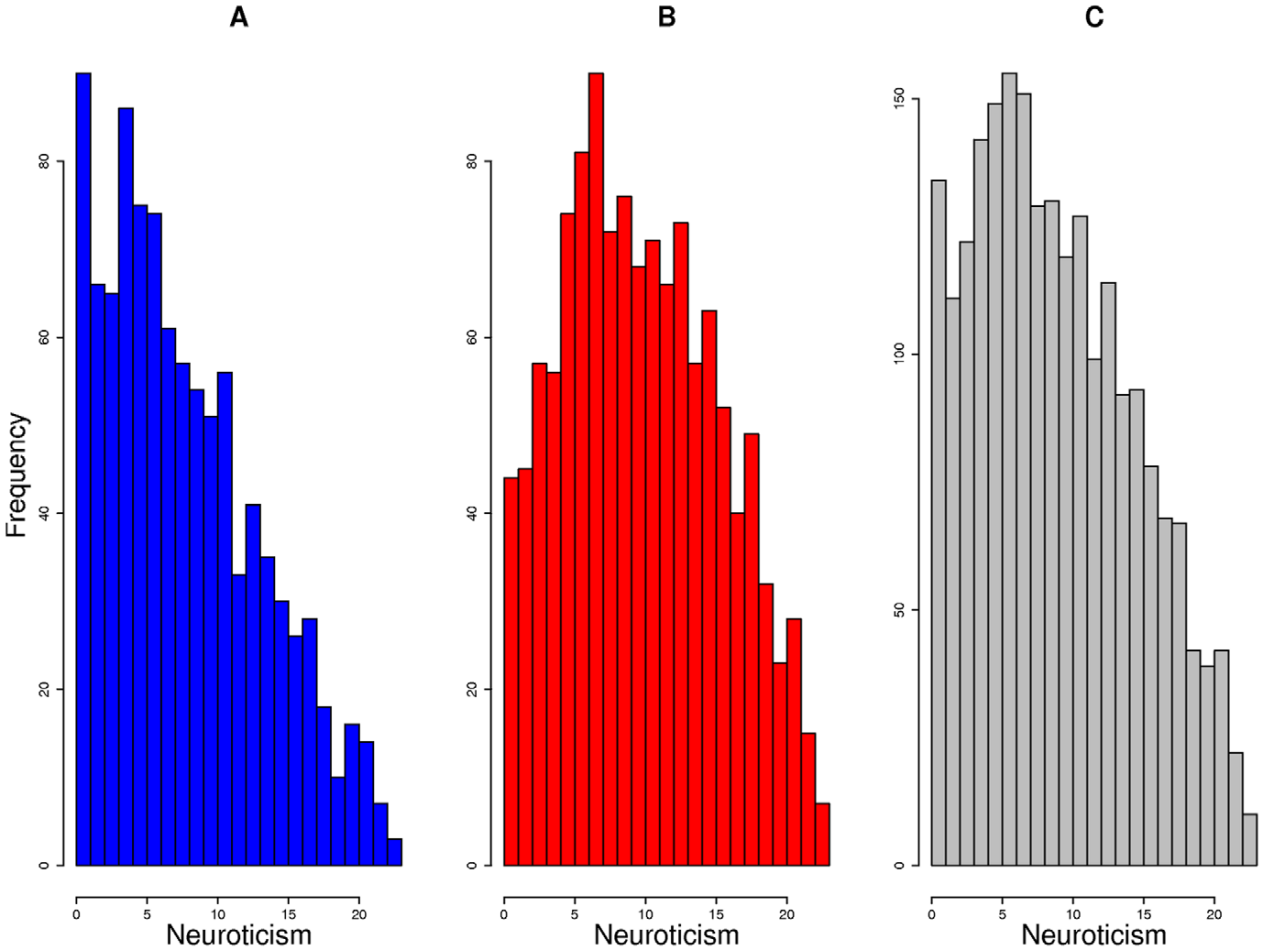
Distribution of EPQ-N score across the subjects. A: Males. B: Females. C: Both sexes.

Neuroticism scores range between 0 and 24, with higher scores indicating more severe neuroticism. See Figure 3 for the distribution of EPQ-N in our study. The overall mean score was 9.33 (SD 5.61), but there was a highly-significant sex difference (males: mean 8.17, median 7, SD 5.50; females: mean 10.26, median 10, SD 5.53; Mann-Whitney test *p*<10^−16^). There was no correlation between age and EPQ-N (r = −0.02, p = 0.26).

### Genotyping and quality controls

Venous blood samples were taken at the time of the participation in the CoLaus study. Nuclear DNA was extracted from whole blood. Participants consented for the genetic data to be used for the study of cardiovascular risk factors and psychiatric disorders. The samples were genotyped on the Affymetrix GeneChip Human Mapping 500K Array Set according to the Affymetrix published protocol. Genotypes were determined by the Bayesian Robust Linear Model with Mahalanobis Distance algorithm (BRLMM). Details of the genotyping procedures are given by Li [35]. A QC procedure was carried out on the sample to remove SNPs with poor amplification and individuals with an excess of missing genotypes. First, a total of 60,545 SNPs with >5% missing genotypes were removed. Next, 4 individuals with missingness >5% among the remaining SNPs were excluded. This left 2,235 individuals genotyped at 430,193 autosomal SNPs. An additional 9,828 SNPs were available on the X chromosome, but only in 2,168 individuals. Overall we flagged 1,893 autosomal SNPs showing significant deviation from HWE (p<10^−4^). None of the SNPs highlighted here were among these 1,893.

### Statistical analysis

Assuming an additive genetic model and a Gaussian-distributed phenotype, 2 235 individuals provide 95% power to detect a genotyped SNP with heritability h^2^ = 0.02 at significance level 5×10^−7^, and 39% power for h^2^ = 0.01. Thus we expect to detect almost all SNPs with h^2^>0.02 and a large proportion of those with h^2^>0.01.

**Figure 4:**
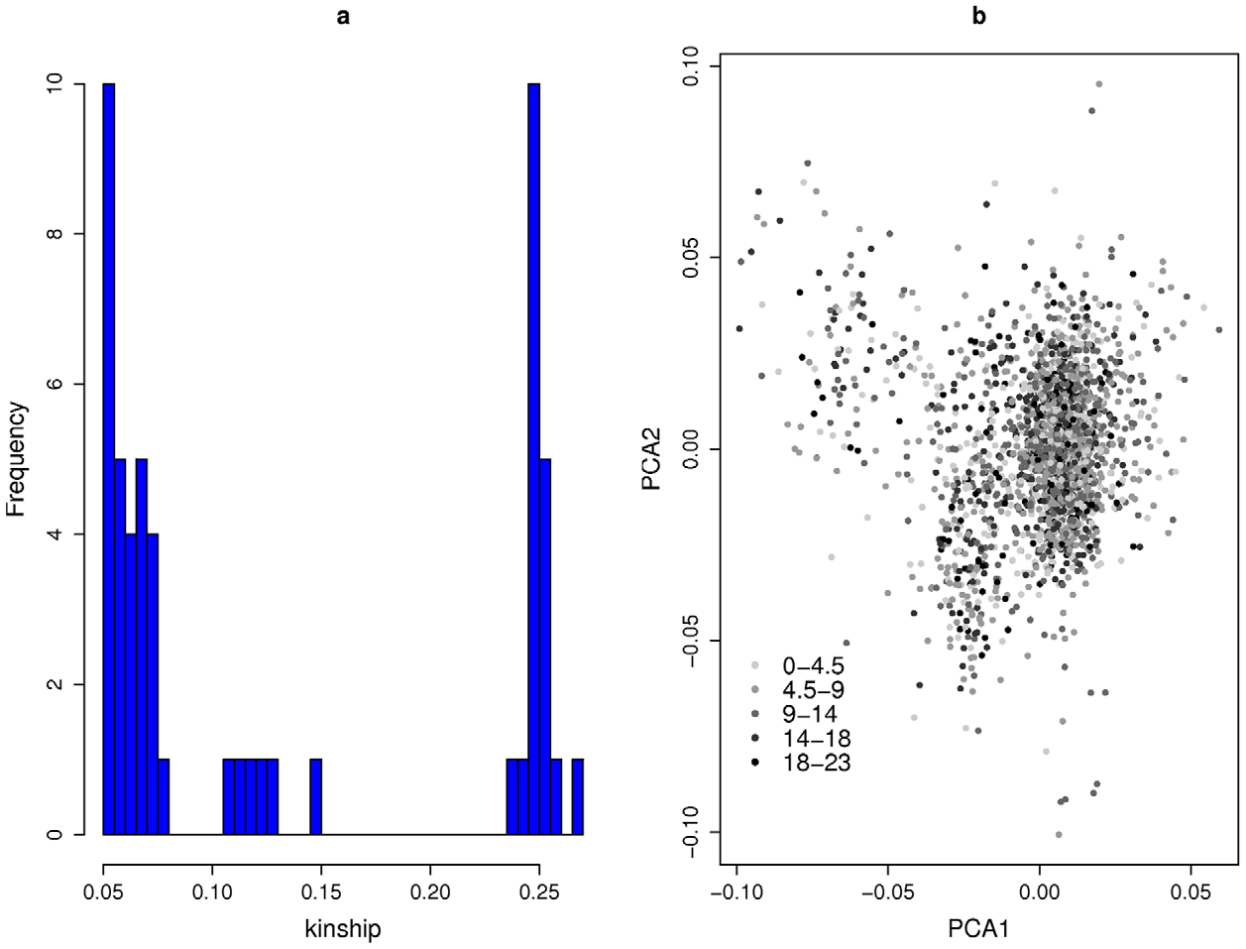
Kinship plots. Plot (a): histogram of kinship coefficients >0.05; Plot (b): Principal component scores obtained from the kinship matrix. Each point represents an individual with EPQ-N score represented by grey scale as indicated on plot.

In order to investigate any possible population structure or cryptic relatedness among our study subjects, we selected a subset of genotyped SNPs to produce a kinship matrix and perform Principal Component Analysis (PCA). The SNPs were chosen using PLINK [36] to have minor allele fraction >5% and pairwise R^2^≤0.5, in a window size of 50 SNPs sliding 5 SNPs at a time. This process chose 75,046 SNPs for kinship and PCA analyses. For these analyses, missing genotypes were replaced with the mean genotype score for that SNP. The kinship matrix is K = XX^T^/2n, where n is the number of SNPs and X is a matrix with rows corresponding to individuals, while each column contains the genotype score for a SNP (i.e. the number of copies of minor allele, 0, 1 or 2), standardised to mean zero and unit variance. The (i, j) element of K is the excess allele sharing for alleles drawn from individuals i and j, beyond that expected for unrelated individuals given the allele fraction estimates [37]. The eigenvectors of K are the principal components, used to diagnose and correct for population structure. The first principal component (PC1) showed some dispersion of individuals away from the main cluster (Figure 4(b)), which may correspond to admixture. This was the only one of the first five PCs that was significantly associated with neuroticism (p<10^−4^), explaining 0.5% of the phenotypic variance. From K we also identified 54 pairs of individuals with kinship estimate >0.05, which corresponds approximately to at least a cousin relationship in an outbred population (Figure 4(a)). Of these 54 kin pairs, 19 had kinships around 0.25, consistent with a first-degree relationship (sibling, or parent/offspring).

To analyse all the study subjects without having to remove one of each kin pair, we considered implementing a mixed-regression association analysis in which phenotypic correlation was proportional to K [38], [39], while age and sex were fitted as fixed effects. The motivation for this model is that any polygenic component of disease causation should be shared among individuals according to their kinships. However, in fitting the mixed model the maximum likelihood estimate of the component of variance attributable to kinship was zero. In fact, among the 19 pairs of apparent first-degree relatives, the correlation in neuroticism was −0.36, which leads to an upper 97.5% confidence limit for the heritability of neuroticism of 0.22. As a consequence of this negative correlation in our study, there is no inflation of test statistics if we ignore kinship and analyse all the study subjects using standard linear regression. After adjusting for age and sex, the EPQ-N score was close normal (Gaussian) and no transformation was necessary.

The genotypes of 2 149 individuals were imputed at over 2 million SNPs using the CEU HapMap samples and the IMPUTE program [40]. We retained for analysis the 1 705 237 genotyped and imputed SNPs with, on average over individuals, probability ≥0.9 assigned to their most likely genotype. These were analysed using a standard linear model implemented in R, with SNP coded as the expected minor allele count. In addition to PC1, age and sex were included as covariates in each model, and the interactions of these terms with the SNP were also tested. The age term used for the linear model was the age at the time of psychological assessment, not that at recruitment.

Finally, we used a Gene Ontology (GO) tree machine (Vanderbilt University) to explore the possible enrichment of any pre-defined functional category among the genes that included a SNP with p<10−4.

## Supporting Information

File S1 LogQQ plot of the p-values of the SNP main effect.
(5.49 MB TIF)
