# Supplementary figures and images for: A Genome-Wide Association Study of Neuroticism in a Population-Based Sample
Source: PLoS One. 2010 Jul 9;5(7):e11504. doi: 10.1371/journal.pone.0011504 (PMC2901337; doi:10.1371/journal.pone.0011504)

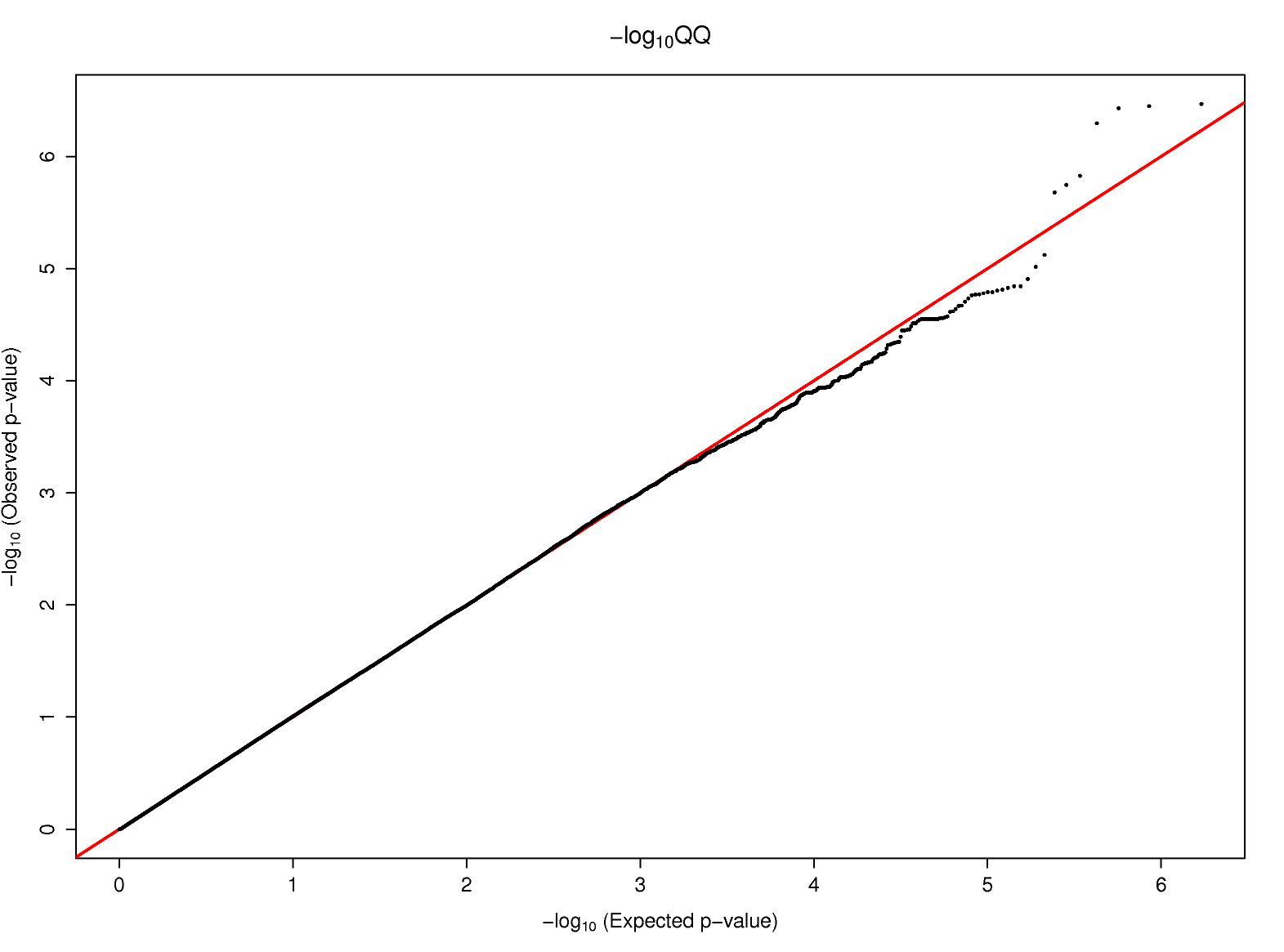

Supplement: File S1 — LogQQ plot of the p-values of the SNP main effect. (5.49 MB TIF) [file pone.0011504.s001.tif]
